# Preparation, *In Vitro* Characterization, and *In Vivo* Pharmacokinetic Evaluation of Respirable Porous Microparticles Containing Rifampicin

**DOI:** 10.3797/scipharm.1307-03

**Published:** 2014-07-23

**Authors:** Aliasgar Kundawala, Vishnu Patel, Harsha Patel, Dhaglaram Choudhary

**Affiliations:** ^1^ Indukaka Ipcowala College of Pharmacy, New Vallabh Vidyanagar, Dist. Anand (Gujarat) – 388121, India.; ^2^ A. R. College of Pharmacy and G H Patel Institute of pharmacy, Vallabh Vidyanagar, Dist. Anand (Gujarat) – 388120, India.

**Keywords:** Microparticles, Chitosan, Inhalation, Sustained release

## Abstract

This study aimed to prepare and evaluate rifampicin microparticles for the lung delivery of rifampicin as respirable powder. The microparticles were prepared using chitosan by the spray-drying method and evaluated for aerodynamic properties and pulmonary drug absorption. To control the drug release, tripoly-phosphate in different concentrations 0.6, 0.9, 1.2, and 1.5 was employed to get a sustained drug release profile. The microparticles were evaluated for drug loading, % entrapment efficiency, tapped density, morphological characteristics, and *in vitro* drug release studies. Aerosol properties were determined using the Andersen cascade impactor. Porous microparticles with particle sizes (d_0.5_) less than 10 μm were obtained. The entrapment of rifampicin in microparticles was up to 72%. *In vitro* drug release suggested that the crosslinked microparticles showed sustained release for more than 12 hrs. The drug release rate was found to be decreased as the TPP concentration was increased. The microparticles showed a fine particle fraction in the range of 55–63% with mass median aerodynamic diameter (MMAD) values below 3 μm. The *in vivo* pulmonary absorption of the chitosan microparticles suggested a sustained drug release profile up to 72 hrs with an elimination rate of 0.010 per hr. The studies revealed that the spray-dried porous microparticles have suitable properties to be used as respirable powder in rifampicin delivery to the lungs.

## Introduction

Drug delivery through the pulmonary route is being explored and studied as a potential non-invasive administration route for the systemic and local delivery of therapeutic agents. Recently, dry powder inhalers (DPIs) have become increasingly popular as a pulmonary delivery system primarily for the following reasons: (a) being in a solid form, the drug is relatively stable chemically; (b) the inhaler is environmentally friendly, as it is free from chlorofluorocarbon (CFC) propellants; and (c) the delivery is breath actuated, and does not require coordination between actuation and inhalation by the patient, as does its pressurized counterparts. The dry powder inhalers deliver a drug in the form of a drug excipient blend or in microparticulate forms like liposomes, nanoparticles, microspheres, or microparticles.

Several studies [1, 2] have demonstrated the clinical advantages of inhalation aerosols over systemic therapy for the treatment of lung disorders. Inhalation drug delivery requires relatively small doses for effective therapy, thus reducing systemic exposure to the drug and minimizing adverse effects. Several researchers suggested the administration of antitubercular drugs in the form of microparticles as inhalable or injectable forms [3–6]. It has been observed that the particles reaching the lungs are phagocytosed rapidly by alveolar macrophages. Although, phagocytosis and sequestration seems to be advantageous in the case of tuberculosis where the drug in a large amount reaches the cytosol effectively [7].

Among the drugs used, rifampicin is the first-line antitubercular drug given orally in combination with isoniazid to treat TB over a period of 4-6 months. The delivery of rifampicin to the lungs in a controlled manner would facilitate the reduction in dose, dose frequency, and toxicity. Delivery of the drug to the lungs serves the purpose of targeting the mycobacterium residing in macrophages [8]. It has been reported that rifampicin shows side effects, notably hepatotoxicity, enzyme induction, renal failure, and drug interactions with several other drugs [9, 10]. The oral dose of rifampicin has difficulty reaching the deep lung where the mycobacterium resides. Since the rifampicin reaches the target tissue through systemic circulation, delivering the drug directly to the lung at a low dose via inhalations seems advantageous with better therapeutic efficacy [11]. Researchers in the filed have prepared and studied the delivery of rifampicin to the lungs as microspheres, pulmospheres, liposomes, and nanoparticles [12–14]. Darbandi et al. suggested the possibility of achieving a minimum inhibitory concentration of the drug in the lungs after studying the pharmacokinetics of inhaled rifampicin in a rat model [15]. Earlier, we demonstrated the effective use of biodegradable polymers like HPMC, HPC, and chitosan in the preparation of inhalable microparticles containing rifampicin [16, 17].

In this study, rifampicin microparticles were prepared by the spray-drying method using chitosan as the release-retarding carrier polymer for the intracellular delivery of rifampicin via inhalation. Tripolyphosphate (TPP) as a crosslinking agent was employed to get a sustained drug release profile from the chitosan microparticles. Chitosan, being a natural, nontoxic, biocompatible, biodegradable with bioadhesive properties, was selected as the carrier polymer.

## Results and Discussion

### Powder Properties of Rifampicin Microparticles

Rifampicin was processed with a biodegradable polymer to prepare the respirable powder for inhalable drug delivery to achieve a better drug therapy for the treatment of tuberculosis. The rifampicin microparticles were prepared by the spray-drying method using two viscosity grades of chitosan (100 cP and 10 cP). The chitosan viscosity was measured by the Brookfield viscometer. The respirable powder was selected as a means of drug delivery to the target lungs considering the associated low oral bioavailability and variability offered by various other dosage forms [18]. The spray-drying technology was effectively utilized in the preparation of the microparticles for the desired physical and aerodynamic particle properties. Up to 49% yield of the spray-dried product (micro-particles) was obtained. It was observed that low viscosity grade chitosan (CL) produced a lesser yield up to 35–39.5% compared to higher viscosity grade chitosan (CH). The lower yield by spray-drying could be attributed to its instrumentation design, the amount of material feed solution, and to the loss of the smallest and lightest particles through the atomization process [19, 20]. Up to 89–98% of the drug loading was obtained by both of the higher viscosity grade chitosan (CH) and lower viscosity grade chitosan (CL) micro-particles. The entrapment efficiency was found to be up to 72%. The entrapment efficiency was further decreased upon the addition of TPP. The decrease in entrapment efficiency could be the lesser binding or incorporation of the drug into the chitosan matrix offered by the addition of TPP. Santos RHT et al. reported that as the amino group of chitosan was unavailable for linking, the interaction of rifampicin with chitosan diminished [21].

### Morphological Characterization

**Fig. 1.**
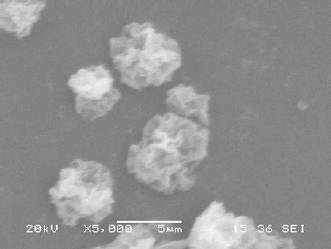
SEM image of TPP crosslinked chitosan microparticles containing rifampicin (CH4)

**Fig. 2.**
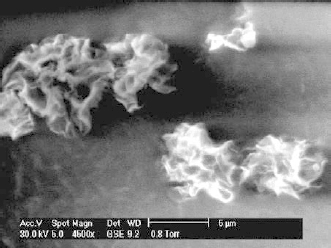
SEM image of TPP crosslinked chitosan microparticles containing rifampicin (CL4)

The morphological studies examined by scanning electron microscopy (SEM) showed porous structured microparticles with irregular shape. The images of the chitosan microparticles under SEM are shown in figures 1 and 2. The microparticles were found to be wrinkled and porous in nature. Patil JS et al. and Darbandi et al. have also reported the development of porous rifampicin microparticles on spray-drying. The depression of the surface polymer to form a wrinkled surface was thought to be the result of faster solvent removal from the surface of the microparticles on drying in the drying chamber at high temperature [15, 22]. The morphological characteristics of the prepared microparticles remained unchanged when a change in chitosan grade or TPP concentration varied.

### Differential Scanning Calorimetry

**Fig. 3.**
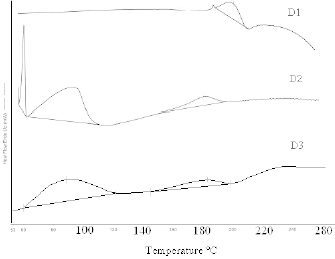
DSC thermogram of pure rifampicin, (D1), rifampicin microparticles CH4, (D2), and CL4, (D3)

The drug excipient compatibility was checked by differential calorimetric analysis. The differential calorimetric studies showed an endothermic peak of pure rifampicin at 189.25°C in the thermogram shown in Figure 3 (D1). The thermograms were obtained by scanning the sample at a rate of 10°C per min. The thermograms of CH4 and CL4, drug-loaded microparticles, showed a peak in the region of 80–125°C and a peak at the valley extending to 240–260°C which indicated the complexation and incorporation of rifampicin in the chitosan matrix. The initial peak at around 80–125°C may be due to the presence of moisture in the microparticles.

The powder properties play an important role in the dry powder inhalation formulation as the deep lung deposition depends mainly on the properties like particle size, shape, morphology, and their densities. The tapped density of the spray-dried powder containing rifampicin was found to be in the range of 0.139–0.162 g/cm^3^ for the CH microparticle and 0.128–0.203 g/cm^3^ for the CL microparticles. Generally, low tapped density powders are considered good for dispersibility and deep lung deposition is governed by the sedimentation mechanism. The lower tapped density of chitosan microparticles was the result of fluffiness produced by the uneven, porous, and wrinkled surface. The particle sizes of rifampicin microparticles were found to be in the range of 4.79–5.40 μm and 5.21–5.70 μm for the CH microparticles and the CL microparticles, respectively. The particle size of the CL microparticles was found to be slightly higher than the CH microparticles. SEM images suggested broadening of the wrinkled strips was seen with the CL microparticles, whereas shrinking of the wrinkled strips was observed in the case of the CH microparticles. The lower water holding capacity, lower viscosity, and faster solvent evaporation at high temperatures may have contributed to the highly irregular shape of the CL microparticles that may have resulted in a larger particle size compared to the CH microparticles. The particle size of the uncrosslinked chitosan microparticles were 5.40 μm and 5.70 μm, whereas the lowest particle size of the crosslinked microparticles were 4.87 μm and 5.21 μm for the CH microparticles and CL microparticles, respectively. The particle size data analysis suggested that the particle size was decreased further with an increase in tripolyphosphate concentration. Similar outcomes were reported by Anal AK where the particle size of the spray-dried chitosan microspheres were found to be decreased with increased TPP concentration [23]. The reduction in particle size after crosslinking with TPP was correlated with lower chain relaxation offered on crosslinking prior to spray-drying.

**Tab. 1.** Composition of spray-dried respirable powders and its powder properties

Batches	% Drug loading	Entrapment efficiency (%)	Tapped density	Particle size (D_0.5_ μm)	Aerodynamic diameter (μm)
CH1	32.37 ± 0.92	70.43 ± 2.8	0.139 ± 0.013	5.40 ± 0.7	1.89
CH2	19.59 ± 0.51	72.18 ± 1.6	0.143 ± 0.018	5.27 ± 0.5	1.95
CH3	19.15 ± 0.23	69.11 ± 2.1	0.147 ± 0.019	4.92 ± 0.4	1.89
CH4	18.95 ± 0.42	68.93 ± 2.1	0.161 ± 0.021	4.87 ± 0.5	1.96
CH5	18.97 ± 0.72	68.23 ± 1.2	0.162 ± 0.028	4.79 ± 0.3	1.92
CL1	19.57 ± 0.63	67.09 ± 1.8	0.128 ± 0.016	5.70 ± 0.7	2.14
CL2	19.36 ± 0.29	69.51 ± 2.8	0.179 ± 0.022	5.53 ± 0.3	2.34
CL3	19.18 ± 0.77	67.94 ± 2.7	0.185 ± 0.016	5.37 ± 0.4	2.31
CL4	19.14 ± 0.82	65.23 ± 1.9	0.189 ± 0.019	5.21 ± 0.5	2.26
CL5	18.33 ± 0.54	65.11 ± 2.2	0.203 ± 0.024	5.46 ± 0.3	2.47

### In vitro Drug Release Studies

Dissolution of the respirable drug once inhaled is critically linked to the onset and duration of its therapeutic activity. The *in vitro* dissolution test was performed using the USP type II apparatus with a slight modification. As expected, the drug release from rifampicin microparticles was found to be slower. The drug release from the chitosan microparticles (CH) showed more delayed drug release compared to the chitosan microparticles (CL).

**Fig. 4.**
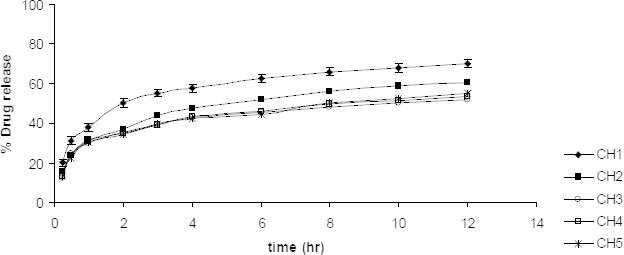
*In vitro* drug release studies of CH formulations in a phosphate buffer of pH 7.4 (n=3)

**Fig. 5.**
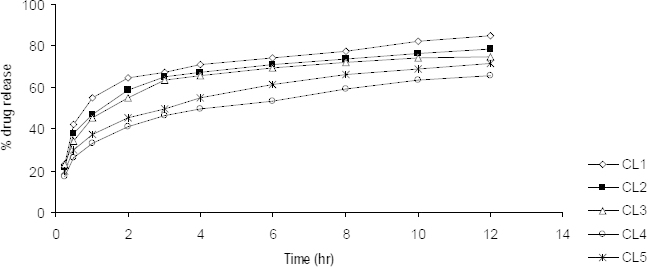
*In vitro* drug release studies of CL formulations in a phosphate buffer of pH 7.4 (n=3)

Uncrosslinked chitosan microparticles were found to release 70% and 86% of rifampicin in 12 hrs for CH and CL microparticles, respectively, in a phosphate buffer of pH 7.4. The drug release was slow and steady from both CH and CL microparticles. TPP-crosslinked CH microparticles were found to release rifampicin up to 60% in 12 hrs, where 0.9% w/v TPP concentration showed the least drug release of 52.07%. Drug release from TPP-crosslinked CL microparticles was quite faster than its counterpart CH microparticles. Drug release was observed to be up to 79%, whereas a comparative delay in drug release was found with the formulation CL4 when 1.2% w/v TPP was employed for crosslinking. The drug release pattern for the CH and CL microparticles are shown in figures 4 and 5, respectively. Addition of the crosslinking agent at various concentrations further reduced the rate of drug release which enabled prolonged release. Although, it would not be appropriate to predict the same drug dissolution and release rate *in vivo*. The conditions like low secretion, large surface area availability, and lung clearance are different from *in vitro* studies.

### Microparticle Respirability

The theoretical aerodynamic diameters (D_ae_) for different batches of microparticles calculated from the tapped density and particle size were in the range of 1.89–2.47 μm, as shown in Table 1. The D_ae_ values were below 5 μm, which indicated effective particle sizes suitable for inhalation. The selected formulations, when subjected to aerosol performance using the Andersen cascade impactor, found that the MMAD values were a little higher than the D_ae_ values. Higher MMAD values could be the result of the aggregation of particles, friction, or interlocking between the particles which might not have disaggregated on aspiration. These MMAD values obtained in the range of 2.60–3.15 μm indicated the suitability of the microparticles to be inhaled into the deep lung. The fine particle fraction (FPF) was up to 62.44% and 58.26% for the CL microparticles and the CH microparticles. The FPF values of the TPP-crosslinked microparticles were found to be increased. Since the particles remained wrinkled and porous after crosslinking with TPP, the FPF was not affected by the little increase in density values. The particle size difference might have also contributed to an increase in FPF values upon increased TPP concentration. The FPF and MMAD are shown in Table 2.

**Tab. 2.** Aerosol properties of spray-dried rifampicin porous microparticles

Formulation	ED (%)	FPF (%)	MMAD (μm)	GSD
CH1	89.41 ± 1.29	55.14 ± 3.11	2.87 ± 0.3	2.06
CH2	91.56 ± 3.42	56.13 ± 2.91	2.68 ± 0.1	2.35
CH3	92.39 ± 1.89	56.27 ± 1.96	2.71 ± 0.2	2.12
CH4	91.72 ± 3.03	58.26 ± 2.49	2.64 ± 0.2	2.01
CH5	92.07 ± 2.54	57.92 ± 2.15	2.60 ± 0.4	2.05
CL1	87.63 ± 2.58	57.47 ± 2.15	2.85 ± 0.2	2.22
CL2	86.25 ± 1.92	60.82 ± 1.51	3.15 ± 0.3	2.62
CL3	88.01 ± 2.12	59.62 ± 2.08	2.96 ± 0.4	2.28
CL4	86.67 ± 1.48	62.23 ± 2.49	2.80 ± 0.2	2.12
CL5	85.47 ± 2.26	62.44 ± 1.83	2.76 ± 0.4	2.15

ED…Emitted dose; FPF…fine particle fraction; MMAD…Mass median aerodynamic diameter; GSD…geometric standard deviation.

The addition of leucine not only produced microparticles with a good yield, but also reflected in good dispersibility which was the result of a decrease in interparticulate cohesive interactions. Leucine was employed as a dispersible agent in the preparation of dry powder inhalers as it coats the particles and improves deaggregation by reducing cohesive forces existing between particles. The other possible reason behind good dispersibility may be due to the porous nature of the microparticles that remain suspended in the air on inspiration. The hydrophobic property of leucine was reported to reduce the moisture content of the microparticles produced by coating the microparticle that in turn improves the dispersibility by reducing cohesiveness [24]. In our previous studies, we reported that the addition of leucine improved the fine particle fraction up to 9% with effective dispersibility that further increased the inhalation by reducing the MMAD values [25].

**Fig. 6.**
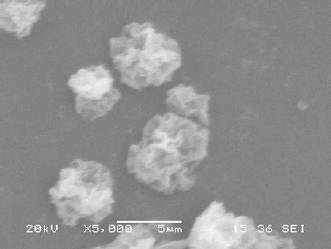
The amount of microparticles deposited on each stage of the cascade impactor plotted against the effective cut of diameter (ECD) on the log probability chart for formulation CH4 (MMAD: 2.64 μm and GSD: 2.01)

The GSD value which is an indication of the dispersibility of particles at different stages of the cascade impactor was found to be in between 2–3, as shown in Table 2. The GSD values indicated towards a monodispersion of particles, suggesting a homogenous distribution with narrow variations. The log probability plot of the cumulative mass distribution of the microparticles collected at various stages in the Andersen cascade impactor is shown in Figure 6.

The biodegradable polymer slow-release microparticle used as respirable powder may possess potential advantages including drug targeting to alveolar macrophages, reduction in dose, and systemic side effects as well as higher patient compliance. A reduction in dosing frequency and other attributes make sustained-release microparticles an alternative for the treatment of tuberculosis, especially for pulmonary tuberculosis.

### Pharmacokinetic Analysis

**Fig. 7.**
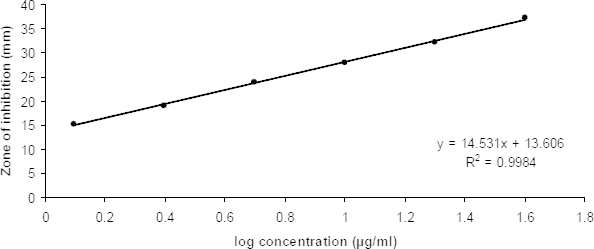
Calibration curve of rifampicin in rat plasma by the disc diffusion method

In this study, the microbial assay method was explored in the detection of rifampicin concentration in plasma. The drug content in plasma was determined quantitatively by a microbiological method using *Staphylococcus aureus* as the indicator organism. The microbiological method offers the benefit of detecting the drug using a smaller blood sample as little as 10 μl. Saito H and Tomioka H have used the microbiological assay method in the determination of rifampicin content in various body organs after the administration of free and liposomal encapsulated rifampicin [26]. Earlier, Joseph et al. have used *Staphylococcus aureus* as a test organism in the detection of rifampicin in blood and urine samples [27]. The calibration of rifampicin was obtained after spiking different drug concentrations in extracted rat plasma. The calibration curve was found to be linear over the range of 1.25–40 μg/ml. The equation of the calibration curve obtained was y = 14.531x + 13.606 with a correlation coefficient (R^2^=0.9984). The calibration curve obtained is shown in Figure 7. The delivery of rifampicin encapsulated in chitosan microparticles was expected to reduce the lung inflammation and lung damage. Intraday variation was found to be in the range of 3.57–1.43% with recovery ranging between 109.43–91.54%. The interday precision was found to be in the range of 1.29–3.53% with recovery in between 112.06–89.35%.

**Tab. 3.** Evaluation of pharmacokinetic parameters

Pharmacokinetic Parameters	Rifampicin (oral)	Rifampicin (insufflation)	CH4	CL4
C_max_ (µg/mL)	4.29	5.92	2.49	3.16
T_max_ (hr)	1	0.5	8	4
AUC_0→t_ (μg.h/mL)	10.49	9.01	105.35	22.45
AUC_0→∞_ (μg.h/mL)	12.01	12.88	211.07	36.58
K_e_ (h^-1^)	0.289	0.271	0.010	0.058
T_1/2_ (hr)	2.4	2.55	65.41	11.80
AUMC (μg.h^2^/mL)	32.13	25.55	3345.3	329.3
MRT (hr)	2.06	2.83	31.75	14.66

**Fig. 8.**
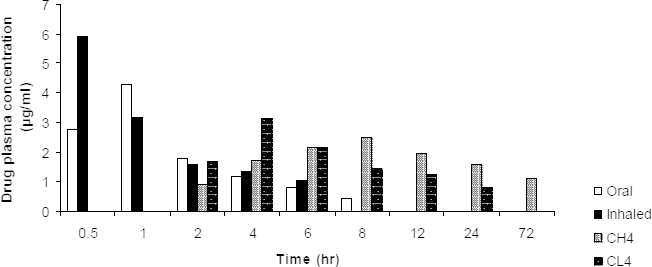
Comparative plasma drug concentration profile of pure rifampicin given by oral and rifampicin pure and microparticles administered by insufflation (CH4 and CL4, n=6)

The various pharmacokinetic parameters, maximum concentration C_max_, time to achieve maximum concentration T_max_, elimination rate constant Ke, half life t_1/2_, area under zero moment curve (AUC_0-72_), and area under first movement curve (AUMC) were derived from the plasma concentration versus time curve and are shown in Table 3. The C_max_ for rifampicin delivered orally was found to be 4.29 μg/ml and C_ma_x for rifampicin delivered via the pulmonary route was found to be 5.92 μg/ml. The faster appearance of rifampicin in blood was attributed to its lipophilicity. Rifampicin was detectable in plasma for 8 hrs in the case of the drug delivered orally, whereas the rifampicin delivered through the pulmonary route showed the presence of the drug for 6 hrs in plasma. The plasma drug concentration was detectable up to 72 hrs after the pulmonary delivery of rifampicin-loaded chitosan microparticles. The plasma concentration profiles of the free drug and selected formulation batches are shown in Figure 8.

The elimination rate after pulmonary delivery was found to be decreased from 0.289 hr^-1^ to 0.010 hr^-1^ for pure rifampicin and the rifampicin formulation (CH4), respectively. The elimination rate for formulation CL4 (0.058 hr^-1^) was a little higher than the formulation CH4 and able to provide more retention in the body compared to the free drug. The elimination half life of rifampicin after pulmonary administration was also found to be increased from 2.55 hr to 65.41 hr indicating a slower drug release from the chitosan microparticles. Sandra S et al. studied the pharmacokinetic behaviour of rifampicin alone and encapsulated with PLGA in mycobacterially infected guinea pigs. They suggested a reduction in inflammation, lung damage, and count of viable bacteria after 28 days of treatment with PLGA microspheres containing rifampicin [28].

## Conclusion

In the present study, the chitosan microparticles containing rifampicin were effectively prepared with suitable aerosol properties to be delivered as inhalable dry powders. The porous- and wrinkled-surfaced microparticles with low tapped density values are thought to be deposited in the lungs by a sedimentation mechanism. The chitosan microparticles showed drug loading up to 98% and entrapment efficiency of 72% that is desirable for effective particular delivery. The slow release rate of rifampicin from TPP-crosslinked microparticles has ensured the sustained release that served the purpose of controlled drug delivery. The fine particle fraction in the range of 65% with MMAD values around 2 μm will help achieve a proper drug fraction in the deep lungs after inspiration of microparticles in the form of inhalable dry powder. The crosslinked CH microparticles containing the drug seem to be able to produce better microparticles for prolonged drug release. The *in vivo* animal studies with TPP chitosan microparticles (CH4) showed a lag time of 2 hrs with a T_max_ of 8 hrs and elimination rate constant of 0.010 hr^-1^, suggesting prolonged drug release. The spray-drying technique has the potential to prepare microparticles using biodegradable polymers with suitable aerosol properties.

## Materials and Methods

Rifampicin (RIF) was obtained as a gift sample from Strides Acrolab, Bamgaluru, India. Chitosan (100 cP) and chitosan (10 cP) were generously provided by the Indian Institute of Fisheries, Cochin, and C E Roeper Gmbh, Hamburg, Germany, respectively. The α-monolactose was generously provided by Meggle, Wasserburg Gmbh and Co., Germany as a gift sample. Ascorbic acid and sodium tripolyphosphate were purchased from S D Fine Chemicals, Baroda, India. Leucine was obtained from Loba Chemicals, India. All other chemicals and solvents used were of analytical grade. *Staphylococcus aureus* (MTCC-7405) was generously supplied by ARIBAS, Anand, India. The experimental protocols were approved by the Institutional Animal Ethics Committee (IAEC), (*Protocol number: IICP/PH/ 02-2010/02*), as per guidelines of the Committee for the Purpose of Control and Supervision of Experiments on Animals (CPCSEA), Government of India.

### Preparation of Spray-Dried Rifampicin Porous Microparticles

Porous microparticles containing rifampicin were produced by the spray-drying method. In brief, rifampicin organic solution was first prepared using ethanol and mixed with 0.75% w/v chitosan solution. Ascorbic acid (200 µg/ml) was added as an antioxidant and the drug to polymer ratio was kept at 1:2. The crosslinking of chitosan was brought about by the dropwise addition of 10 ml of sodium tripolyphosphate (TPP) solution at various concentrations (0.6, 0.9, 1.2, and 1.5% w/v). The mixture was kept on stirring at 1000 rpm for 30 min. The lactose and leucine (ratio of 1:3) in the required quantity was added into the mixture. The prepared suspension was then spray-dried using a 0.7 mm standard nozzle at 150ºC, 5 ml/min feed rate, and 2.5 kg/cm^2^ of pressure. The liquid feed volume was kept at 300 ml for all of the formulation batches. The dried product in the form of powder was collected from a cyclone separator and kept in a desiccator until further use.

### Characterization of Spray-Dried Microparticles

#### Scanning Electron Microscopy (SEM)

The surface morphology and shape of the microparticles were examined by means of electron microscopy. The spray-dried powders were mounted onto separate, adhesive-coated 12.5 mm diameter aluminum stubs. Excess powder was removed by tapping the stubs sharply and then gently blowing a jet of particle-free compressed gas across each. The specimens were examined under the electron microscope. The SEM was operated at a high vacuum with accelerating voltage of 5-15 KV and a specimen working distance of 12 mm.

#### Drug Loading

The drug loading capacity of the spray-dried powder was obtained by the determination of drug content from the lysed microparticles. The required quantity (25 mg) of microparticles was dissolved 0.1 N HCl. The drug content was determined by taking the absorbance at 475 nm in UV spectrophotometer after the suitable dilution. The loading was then expressed as mg of drug/mg of microparticles.

#### Drug Entrapment Efficiency

The % entrapment efficiency of the microparticles was calculated by extracting the drug from the microparticles (25 mg) in a phosphate buffer, pH 7.4, containing ascorbic acid (200 μg/ml). The microparticles were first dispersed in the phosphate buffer at pH 7.4 and subjected to centrifugation at 5000 rpm for 15 min. The supernant was collected and filtered through a 0.45 µm membrane filter. The filtrate was assayed for drug content by UV spectrophotometry at the wavelength of 475 nm. The percentage entrapment efficiency for each drug was calculated by the formula:


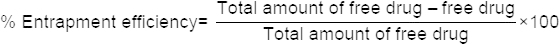


#### Particle Size Determination

The particle size of the spray-dried powder was measured by the laser diffraction method. The particle size was measured by the HELOS particle size analyzer VIBRO/RODOS dry dispersion system: Sympatec Gmbh System Partikel Technik, Germany, equipped with a computer-controlled image analysis system. Approximately 100 mg of the powder was used to achieve the required obscuration of 5%. The particle size data obtained were represented as D_0.5_.

#### Differential Calorimetric Analysis

Differential scanning calorimetry (DSC-PYRIS-1, Perkin Elmer, USA) was used to investigate the effect of the incorporation of the drug on the chitosan matrix. The samples were heated from 50 to 300*°C* at a scanning rate of 10°C /min.

#### Powder Densities and Aerodynamic Diameter

The powder density was evaluated by a tapped density measurement. Densities of the microparticles were determined using a 10-ml measuring cylinder after filling the known mass (0.5 g) of powder under gravity and recording the volume occupied by the powder. The tapped densities of all the formulations were determined by tapping the measuring cylinder from a constant height, and the volume of the tapped mass was noted until no further change in the powder volume was observed. Measurement was performed in triplicate (n = 3).

The theoretical estimation of the particle primarily aerodynamic diameter MMAD_t_ was derived from the sizing (D_0.5_) and tapped density data (ρ) according to following equation [29]:


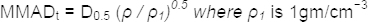


#### Andersen Cascade Impactor

The actual aerodynamic diameter and the aerosol performance of the formulations were tested by the eight-stage Andersen cascade impactor (ACI). The ACI consists of an induction port, pre-separator, seven stages, and a final filter. The pre-separator was attached to the impactor to prevent large particle aggregation. After assembling the ACI stages, the assembly was then attached to a vacuum pump, equipped with a flow meter. The air flow was than adjusted for 60 L/min [30]. Capsules (HPMC, size 2) were filled with powder containing 3 mg of rifampicin and 10 capsules were subjected for discharge into the apparatus per determination. The powder was washed with 0.1 N HCl from each stage and analyzed for drug content. The drug content was determined by UV spectrophotometry at a wavelength of 475 nm. The effective cutoff diameters obtained for stages 0-6 were 6.5, 4.4, 3.2, 1.9, 1.2, 0.55, and 0.26 µm [31].

The fine particle fraction (FPF) of the total dose of powder less than 5 µm was calculated by dividing the powder mass recovered from the stages of the apparatus by the total mass emitted. The cumulative mass of the powder less than the stated size of each stage was calculated and plotted on a log probability scale as a % total mass recovered from the apparatus against the effective cutoff diameter. The mass median aerodynamic diameter (MMAD) was derived from the graph of cumulative distribution as the particle size at which the line crosses the 50% mark.

#### In vitro Drug Release Studies

The *in vitro* release of rifampicin from the spray-dried microparticles was determined by the USP type II dissolution apparatus employing a dialysis membrane (cutoff diameter 12-14 K Daltons) as described in our previous studies [17]. In brief, the dissolution test was performed placing 50 mg of rifampicin in a pre-soaked dialysis membrane bag (size, 3 × 5 cm) and clipped to a paddle of the dissolution apparatus, rotating at speed of 150 rpm. The phosphate-buffer solution at pH 7.4 (900 ml) containing 200 μg/ml of ascorbic acid was employed as dissolution media. The test samples were withdrawn at predetermined time intervals and the drug content was determined by measuring absorbance by a UV spectrophotometer at a wavelength of 475 nm. An equal volume of fresh dissolution medium was replaced immediately after the withdrawal of each test sample. The cumulative percentage drug release was calculated and plotted against time. Each dissolution experiment was performed in triplicate.

### Pharmacokinetic Analysis

#### Experimental

Wistar albino rats (250–300 g) were used for the animal experiments. Rats were housed in a room maintained on a 12 hr light/dark cycle at 23 ± 2°C with free access to food and water. The animal experimental procedures were approved by the Institutional Animal Ethics Committee (IAEC). The animals were divided into five (5) groups, each group containing 6 animals (n=6). One group was kept as the control. Animals of group 2 and group 3 received the pure drug (5 mg/kg) via the oral route and pulmonary route (insufflation) in a suspension and dry powder form, respectively. The other two groups were insufflated with selected drug formulations (CH4 and CL4) via the pulmonary route (5 mg/kg). The rats fasted for 1 day before the experiments and kept on water intake only. The rats were anesthetized by intraperitoneal injection of 50 mg/kg ketamine: 5 mg/kg xylazine.

The powder formulation (5 mg/kg ~1.25 mg/dose) was insufflated using 3 ml of air by insufflator syringe (insufflator prepared in laboratory). The powder was introduced to the rat lung which was maintained at an angle of 80°. The drug solution in PBS (1 mg/0.5 ml) was orally administered by an oral feeding needle. After the administration of the formulations, the rats were positioned to an angle of 10°C. The volume of the dose and air in the syringe were kept constant in each case. Serial blood samples were collected periodically by puncturing of retro-orbital plexus using heparinzed capillaries and collected in heparinzed centrifugation tubes with aseptic precautions under mild ether anesthesia. Blood samples of 1 ml volume were collected at pre-specified time intervals (at 0.5, 1, 2, 4, 6, 8, 12, 24, and 72 hrs) and cold centrifuged for 30 minutes at 5000 rpm below 4°C. The supernant plasma was collected and a paper disk of 8 mm in diameter was immersed into the supernant plasma. The disc was then placed on an agar plate which was previously inoculated with *Staphylococcus aureus* and kept incubating for 24 hrs at 37°C ± 2°C. The zone of inhibition was measured by a digital vernier caliper and the concentration was calculated from a standard curve.
